# Beyond Radical Scavengers: Focus on NADPH Oxidases (NOX) Inhibitors as New Agents for Antioxidant Therapy in Alzheimer’s Disease

**DOI:** 10.3390/antiox15010017

**Published:** 2025-12-22

**Authors:** Leonardo Brunetti, Fabio Francavilla, Angela Santo, Daniele Vitone, Marcello Leopoldo, Enza Lacivita

**Affiliations:** Dipartimento di Farmacia—Scienze del Farmaco, Università degli Studi di Bari Aldo Moro, Via Orabona 4, 70125 Bari, Italy; fabio.francavilla@uniba.it (F.F.); angela.santo@uniba.it (A.S.); daniele.vitone@uniba.it (D.V.); marcello.leopoldo@uniba.it (M.L.); enza.lacivita@uniba.it (E.L.)

**Keywords:** Alzheimer’s Disease, oxidative stress, neuroinflammation, neurodegeneration, NADPH oxidase, antioxidant therapy

## Abstract

Over the past decade, oxidative stress and neuroinflammation have been increasingly recognized as part of the pathology of Alzheimer’s disease (AD). This observation has led to extensive efforts and attempts to apply antioxidant compounds as therapeutic agents for AD and other pathologies. However, most, if not all, of these attempts have failed in preclinical or clinical trials. A tentative explanation for this failure is radical scavengers’ intrinsic lack of specificity in either their mode or district of action. The lack of specificity has been thought by some to be a source of so-called “reductive stress”, another form of redox imbalance that might be just as toxic as oxidative stress. Thus, research interest is shifting from developing simple radical scavengers to designing and refining compounds targeting the overproduction of Reactive Oxygen Species (ROS) in specific pathological conditions. This can be achieved, for instance, by targeting the enzymes that are mainly responsible for their production, namely NADPH oxidases (NOX). In this review, we will discuss, from the point of view of medicinal chemistry, the main innovations in the development of NOX inhibitors and their potential employment for AD therapy. We will also discuss the experimental hurdles that slow down research in this field and possible solutions.

## 1. Introduction

Neurodegenerative diseases (NDs) are conditions that cause the gradual loss of brain function, resulting in cognitive deficits and motor impairments. In the most severe cases, they can lead to death. Among NDs, Alzheimer’s disease (AD) is the most common. It is characterized primarily by disturbances to memory and executive function. There are two main organic correlates to AD: senile plaques composed of amyloid-beta aggregates (Aβ) and neurofibrillary tangles (NFTs) composed of hyperphosphorylated tau. These structures interfere with synaptic connections, triggering neuronal degeneration and the progressive loss of cerebral tissue [1,2].

Despite comprising only ~2% of total body mass, the brain consumes nearly 20% of the body’s oxygen, rendering neurons, cells with high metabolic demand, particularly susceptible to oxidative stress and ROS-mediated injury [3]. Recent evidence suggests that, while not inherently neurotoxic, amyloid-β (Aβ) peptides indirectly induce neuronal dysfunction via the activation of microglia and astrocytes. This leads to the release of pro-inflammatory cytokines, nitric oxide (NO) and ROS, thus amplifying neuroinflammation and significantly contributing to the oxidative damage seen in Alzheimer’s disease [4,5,6].

The present review aims to explore the impact of oxidative stress on the pathogenesis of neurodegenerative diseases, with a particular focus on the role of NADPH oxidases (NOX). We will examine the main molecular pathways involved, as well as the potential therapeutic implications of modulating the activity of these enzymes to counteract the progression of AD, and the most recent developments in terms of NOX inhibitors.

### 1.1. ROS and the Aging Process in the Central Nervous System

In the mid-1950s, Denham Harman introduced the free radical theory of aging. This theory remains a widely accepted model for some of the molecular mechanisms underlying aging [7]. According to this theory, the progressive accumulation of ROS damages key cellular macromolecules, including lipids, proteins, and nucleic acids. As endogenous antioxidant defenses become less effective with age, an imbalance between ROS production and neutralization contributes to the onset of cellular dysfunction and age-related diseases [7,8].

Aging affects multiple organ systems, notably impacting the immune, cardiovascular, and central nervous systems (CNS) [9]. In the CNS, aging is associated with telomere shortening, DNA damage, mitochondrial dysfunction, and chronic low-grade inflammation [10]. As age advances, the expression of anti-inflammatory cytokines, such as interleukin-10 (IL-10), decreases, while the expression of pro-inflammatory cytokines, such as TGF-β, TNF-α, and IL-1β, increases. TGF-β, although primarily a regulatory cytokine with anti-inflammatory functions, can also become dysregulated in aging and contribute to maladaptive responses in specific CNS contexts. This exacerbates neuroinflammation. Chronic activation of microglia and astrocytes contributes to these inflammatory responses, disruption of the blood–brain barrier (BBB), neuronal degeneration, and increased ROS production. This creates a vicious cycle that accelerates neuronal dysfunction [11,12,13,14].

### 1.2. The Mechanisms of Oxidative Stress and Its Cellular Impact

While low levels of ROS are essential for normal physiological signaling, high levels cause oxidative stress and widespread damage to cellular structures [15]. An increase in ROS leads to oxidative damage in cellular compartments and molecules, including the plasma membrane. There, lipid peroxidation occurs, resulting in the formation of toxic aldehydes, such as 4-hydroxy-2-nonenal (HNE). HNE is detectable in the early stages of disease and its levels are proportional to the extent of neuronal damage [16,17]. Oxidative stress occurs when ROS production overwhelms cellular antioxidant systems or when these systems lose efficiency, which often happens during aging. ROS are byproducts of mitochondrial respiration and other metabolic pathways. Free radicals, which present one or more unpaired electrons, are formed as intermediates in the gradual reduction in molecular oxygen to water [18]. A key event in ROS-induced damage is the formation of hydroxyl radicals via the Fenton reaction, in which hydrogen peroxide is converted into the highly reactive hydroxyl radical (^•^OH) species [19].

Hydroxyl radicals can damage lipids, carbohydrates, proteins, and DNA. Phospholipids, which are abundant in neuronal membranes and rich in polyunsaturated fatty acids (PUFAs), are particularly vulnerable to oxidative damage. This results in the production of reactive byproducts, such as malondialdehyde (MDA) and 4-hydroxynonenal (4-HNE). These byproducts are elevated in Alzheimer’s disease and mild cognitive impairment [20]. Similarly, protein oxidation and nitration result in structural and functional alterations. Oxidative modifications of DNA and RNA, on the other hand, contribute to genomic instability and early neuronal dysfunction [21,22,23].

### 1.3. Oxidative Stress in Dementia and Alzheimer’s Disease

The generation and accumulation of reactive oxygen species (ROS) is a multifactorial phenomenon involving intracellular sources, such as mitochondria, and membrane-bound enzymes, such as NADPH oxidases (NOX). Microglia can particularly amplify oxidative stress through an oxidative burst controlled by the translocation of the regulatory subunits p47^phox^ and p67^phox^ [15]. In AD, oxidative stress is a crucial mechanism strongly intertwined with microglial dysfunction and NOX activation. In addition to causing structural damage, ROS act as intracellular messengers that can activate signaling pathways involved in the pathogenesis of AD. One relevant example is the activation of p38 MAPK during amyloid-β-mediated oxidative stress [15].

In primary neuronal models, this activation is associated with tau phosphorylation. This event can be prevented by treatment with a specific p38 inhibitor or vitamin E [24]. Additionally, research on the role of microglia has revealed that these cells, once considered merely “resident macrophages,” are dynamic mediators of neuroinflammation and regulators of the neuroimmune balance. In AD, microglia surround amyloid plaques and respond to amyloid-β by adopting a pro-inflammatory phenotype characterized by the expression of cytokines such as IL-1β, IL-6, and TNFα. However, microglia are not limited to a single state; they can assume a range of phenotypes, from protective homeostatic functions to harmful responses [25,26,27]. This plasticity explains the dual role of microglia: they contribute to the defense of neuronal tissue, but if chronically activated, they support neurodegenerative processes.

Among the effector mechanisms, ROS production plays a central role. Like peripheral phagocytes, microglia can also undergo an ‘oxidative burst’ in response to damage stimuli, aggregated proteins or cellular debris [28,29]. However, if ROS production exceeds the brain’s antioxidant capacity, which is limited due to its modest defenses and restricted regenerative capacity, oxidative stress is established, contributing to neuronal loss [30,31,32]. Direct evidence of this process comes from experimental studies analyzing the localization of NADPH oxidase components in the AD brain. For the first time, it was demonstrated that the cytosolic factors p47^phox^ and p67^phox^, which are normally located in the cytosol under resting conditions, translocate markedly to the membrane in areas of the brain affected by AD, strongly suggesting the activation of NADPH oxidase. As the antibodies used showed non-specific reactivity, the authors resorted to using primary cultures of rat neurons, astrocytes, and microglia [33]. The results confirmed that p47^phox^ and p67^phox^ are only expressed in activated microglia, and that the translocation of these subunits is necessary for the assembly of the oxidase complex and the production of ROS [34].

Activation of NADPH oxidase results in the formation of superoxide anion (O_2_^•−^), which rapidly converts into secondary reactive species, including hydrogen peroxide (H_2_O_2_), hydroxyl radicals (^•^OH), hypochlorous acid (HOCl), and reactive nitrogen species (RNS), such as peroxynitrite, through interaction with nitric oxide. These diffusible molecules can propagate damage at a distance, contributing to the oxidative neuronal damage observed in AD [35]. Based on these data, attention has focused on the activation of different NOX isoforms in the brain of patients with AD. NADPH oxidases are a major source of ROS under pathological conditions, and their activation is considered a pivotal event in chronic neurodegeneration. In particular, the translocation of the p47^phox^ and p67^phox^ subunits to the membrane directly indicates NOX2 activation [33].

Activation of this isoform in microglia is considered a major source of ROS associated with disease progression [36]. Concurrently, increased expression of NOX1 and NOX3 in the early stages of AD has been associated with markers of mitochondrial dysfunction [37], indicating a synergistic relationship between enzymatic and mitochondrial oxidative stress. Post-mortem studies supporting this hypothesis have shown a correlation between cognitive impairment observed in AD patients and NOX activity measured in the frontal and temporal cortices [38]. Finally, damage-associated molecular patterns (DAMPs), which are released by damaged or dying cells, perpetuate the inflammatory and oxidative cycle. Molecules such as amyloid-β, fibrinogen, HMGB1, TFAM, and cytochrome c stimulate the phagocytic activation of microglia via damage recognition receptors such as complement receptor 3 (CD11b/CD18) and toll-like receptor 4 (TLR4), thereby enhancing ROS production and neuroinflammation [39].

Taken together, these findings demonstrate that the activation of NADPH oxidase, particularly NOX2 in microglia, together with NOX1 and NOX3, is closely associated with ROS and RNS production, oxidative damage and neuronal loss. This manifests clinically as cognitive decline [33,37,40,41].

### 1.4. Reductive Stress: The Paradox of Antioxidant Therapy

While the previous paragraphs illustrated how the balance between ROS production and antioxidant capacity influences neurodegeneration, recent evidence has highlighted a seemingly paradoxical phenomenon: excessive accumulation of reducing equivalents (reductive stress) can precede and contribute to oxidative stress [42]. Studies in human embryonic kidney cells have shown that this reductive state can subsequently trigger an increase in ROS in vitro, suggesting a dynamic transition between reduction and oxidation.

The p38 MAP kinase, which is activated by oxidative stress, plays a central role in the pathophysiology of many neurodegenerative diseases, including AD [43,44,45]. Its activation is closely linked to inflammation and tau phosphorylation, both of which contribute to neurodegeneration. Studies of healthy individuals at risk of AD, the descendants of patients carrying the ApoE4 genotype, have found that two markers of oxidative stress (p38 phosphorylation and glutathione oxidation) are paradoxically decreased compared to controls, despite being elevated in patients with AD [44,46]. This suggests that the activation of antioxidant systems in young individuals may generate a state of reductive stress that is exhausted in later years, facilitating the onset of oxidative stress and cellular damage.

As access to brain tissue is limited, studies have utilized peripheral lymphocytes as a surrogate model to demonstrate that the observed changes in these cells reflect alterations in the brain associated with the disease [46,47]. Lymphocytes from AD patients exhibit increased levels of P-p38 and oxidized glutathione, which are related to oxidative damage and altered protein metabolism [48]. By contrast, healthy individuals at risk exhibit lower levels of these markers, accompanied by the overexpression of antioxidant enzymes such as glutamyl-cysteinyl ligase. This suggests an adaptive response that may precede the onset of the disease [46]. This paradoxical effect is consistent with observations in animal models and human imaging studies. Young, cognitively normal ApoE4 carriers show increased cerebral glucose consumption and activation of parietal and prefrontal regions during memory tasks; these phenomena are related to subsequent cognitive decline [49,50,51]. These findings suggest that reductive stress may coincide with periods of metabolic and neural hyperactivation, acting as a precursor to the oxidative stress that characterizes full-blown AD.

At the molecular level, cellular redox pairs such as NAD(H), NADP(H) and GSH/GSSG regulate energy metabolism and antioxidant capacity. Their excessive accumulation can lead to reductive stress by providing excess electrons for the generation of mitochondrial ROS and activating mechanisms of cellular dysfunction [52,53,54,55,56,57]. Similarly, NADPH fuels NOX enzymes in producing O_2_^•−^ and H_2_O_2_, while GSH supports peroxidases in reducing peroxides [52,53,58,59]. Excess NADPH and GSH, as observed in cardiac models, can paradoxically increase ROS production, generating reductive and oxidative stress and resulting in tissue damage [60]. In summary, reductive stress is an initial and compensatory stage in cellular redox dynamics. If this stage is prolonged or exhausted, it can trigger an outbreak of oxidative stress. This process is closely associated with the production of ROS, oxidative damage and the progression of neurodegeneration, thereby linking early metabolic alterations with the cognitive decline observed in individuals at risk of AD.

### 1.5. Reductive Stress and NADPH/GSH-Driven NOX Activation

While reductive stress is initially characterized by an excess of reducing equivalents, this biochemical state can directly promote oxidative damage through the activation of enzymatic ROS sources. NADPH oxidases (NOX) are key mediators of this process, as they use NADPH to generate superoxide (O_2_^•−^) and hydrogen peroxide (H_2_O_2_).

Experimental models demonstrate that perturbations in NADPH and GSH pools modulate NOX-dependent ROS production.

Cardiac-specific overexpression of wild-type NOX4 (WT-NOX4) decreases NADPH/NADP^+^ and GSH/GSSG ratios and markedly elevates ROS production and cardiac dysfunction following ischemia/reperfusion (IR) injury. Conversely, overexpression of a dominant-negative NOX4 mutant (DN-NOX4), which lacks oxidase activity, increases NADPH and GSH pools but still results in elevated ROS and cardiac damage under IR challenge, indicating that excessive reducing equivalents can enhance ROS production both through NOX-dependent and NOX-independent mechanisms [60].

Complementary evidence comes from metabolic manipulation of the NADPH pool: overexpression of glucose-6-phosphate dehydrogenase (G6PD), the main cytosolic generator of NADPH, increases NADPH levels, upregulates NOX cofactors, and amplifies ROS generation in pancreatic β-cells and thymic lymphoma cells, whereas G6PD deficiency limits reductive stress and attenuates ROS formation [61,62].

**Figure 1:**
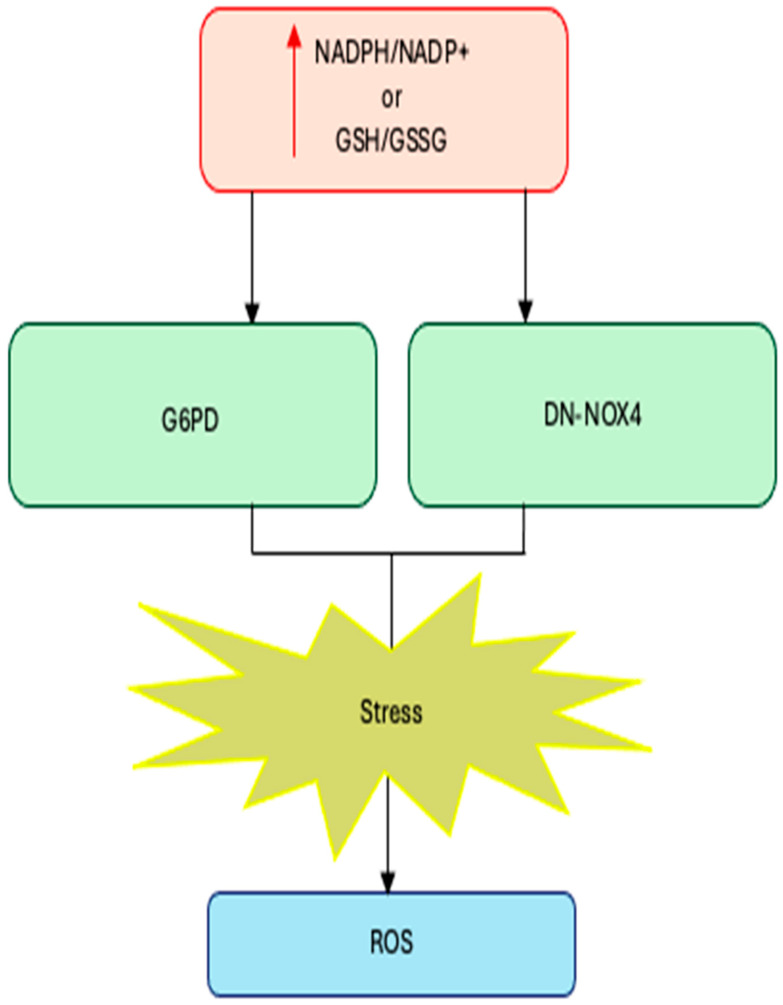
Excess NADPH or GSH levels trigger reductive stress. Increases in NADPH/NADP+ or GSH (GSSG due to increased production (G6PD overexpression) or decreased consumption (DN-NOX4 overexpression) lead to reductive stress.

Together, these findings establish a mechanistic link between reductive stress and enzymatic ROS production: when NADPH and GSH pools are excessively expanded, NOX enzymes become potent amplifiers of oxidative stress, converting a compensatory reductive state into a pathological oxidative one (Figure 1).

### 1.6. Clinical Evidence on the Limitations of Antioxidant Therapy

The mechanistic insights from preclinical studies are mirrored by clinical data highlighting the limited efficacy—and in some cases the potential risks—of indiscriminate antioxidant supplementation. According to the 2022 US Preventive Services Task Force (USPSTF), β-carotene and vitamin E supplements should not be used for the prevention of cardiovascular disease or cancer in healthy adults, as potential harms outweigh any proven benefit [63].

A 2022 meta-analysis of ten randomized trials (~182,800 participants) reported that β-carotene supplementation failed to reduce cardiovascular events and was associated with a slight increase in cardiovascular mortality (RR ~1.12) and incidence (RR ~1.04) [64].

These findings provide a coherent explanation for why antioxidant trials often fail: when reducing equivalents such as NADPH and GSH are already elevated, further global antioxidant supplementation may be ineffective or even detrimental by exacerbating reductive stress and fueling enzymatic ROS sources such as NOX. This convergence of preclinical and clinical evidence supports a shift toward therapeutic strategies that target specific ROS-generating pathways, rather than relying on nonspecific antioxidant supplementation.

## 2. NOX & DUOX Physiopathology

### 2.1. Role of NOX/DUOX in Oxidative Stress

NADPH oxidases (NOX) and dual oxidases (DUOX) represent the only enzyme families whose primary function is the deliberate generation of ROS. In the CNS, ROS serve a dual role: at moderate levels, NOX/DUOX-derived ROS act as second messengers in synaptic plasticity, neurotransmission, and immune signaling; under pathological conditions, however, ROS sustained activity drives oxidative stress, neuroinflammation, and neuronal damage [15,65,66]. Distinct isoforms display specific structural properties, regulatory mechanisms, and cellular localizations, which collectively determine their contribution to disease pathogenesis.

While moderate ROS are essential for baseline cellular signaling, chronic activation of NADPH oxidase 2 (NOX2) has been shown to trigger a condition known as “initiating oxidative stress” (iOS) which plays a key role in amyloid-β (Aβ_1–42_)–mediated neuronal dysfunction [67,68,69]. Given the specificity limitations of conventional antioxidants, including their potential to cause reductive stress [70,71], there is growing interest in the development of isoform-targeted NOX inhibitors as promising therapeutic approaches for neurodegenerative diseases.

### 2.2. NOX/DUOX Family Architecture and Activation

The NOX family, comprising five isoforms (NOX1–5) and two dual oxidases (DUOX1 and DUOX2), is a group of membrane-bound heme-flavin oxidases involved in physiological and pathological ROS production. Each isoform has distinct structures, tissue distributions, and activation mechanisms that define their roles in cellular redox biology (Table 1).

NOX1, NOX2, and NOX3 require the assembly of a multi-subunit complex consisting of the membrane-bound subunit p22^phox^ and several cytosolic regulators, such as p47^phox^, p67^phox^, NOX organizer 1 (NOXO1) and NOX activator 1 (NOXA1) [58,72]. These isoforms are typically activated in response to extracellular stimuli, including pro-inflammatory signals, and are tightly regulated at multiple levels. In contrast, NOX4 is constitutively active and produces ROS in a more sustained manner independently of cytosolic regulators. This feature makes NOX4 particularly relevant in chronic conditions, where prolonged ROS signaling contributes to tissue damage [73]. NOX5, DUOX1, and DUOX2 are structurally distinct in that they contain EF-hand calcium-binding domains, which regulate their enzymatic activity through Ca^2+^ binding and phosphorylation. Additionally, DUOX1 and DUOX2 require specific maturation factors (DUOXA1 and DUOXA2) to ensure their correct trafficking from the endoplasmic reticulum to the plasma membrane [74,75]. Although primarily expressed in epithelial tissues, these enzymes have gained attention for their emerging role in inflammation and neurodegeneration.

**Table 1:** Structural characteristics, activation mechanisms, and cellular localization of NOX and DUOX isoforms.

Isoform	Structural Features	Activation Mechanism	Cellular Localization
NOX1	Forms a membrane-bound complex with p22^phox^ and requires cytosolic subunits (p47^phox^, p67^phox^, NOXO1, NOXA1) [58,72]	Activated by extracellular stimuli via cytosolic subunit recruitment and RAC1-dependent signaling [76,77]	Predominantly in colonic epithelium and vascular cells; inducible in neurons [75,78]
NOX2	Composed of gp91^phox^–p22^phox^ heterodimer with cytosolic subunits (p47^phox^, p67^phox^, p40^phox^, RAC1/2) [79,80]	Upregulated under NF-κB-mediated inflammatory signaling; assembles upon immune stimuli [79,80]	Widely expressed in microglia, endothelial cells, and phagocytes [81,82,83,84]
NOX3	Structurally similar to NOX1; associates with p22^phox^ [85,86]	Largely constitutively active; modulated by p22^phox^ [85,86]	Inner ear (vestibular system), fetal spleen and kidney [85,86]
NOX4	Constitutively active isoform, tightly associated with p22^phox^; does not require cytosolic subunits [73]	Generates sustained H_2_O_2_ independently of external stimuli [73]	Ubiquitously expressed; high levels in kidney, vascular smooth muscle, endothelium, neurons, and astrocytes [58,87]
NOX5	Unique EF-hand calcium-binding motifs; does not require p22^phox^ [88]	Activated by intracellular Ca^2+^ binding and phosphorylation [88]	Testis, spleen, lymphoid tissues (absent in rodents); potential expression in human brain models [88]
DUOX1	Contains extracellular peroxidase-like domain and intracellular EF-hand motifs; maturation depends on DUOXA1 [89]	Requires Ca^2+^ binding and DUOXA1 for ER exit and membrane targeting [89]	Thyroid and epithelial tissues; upregulated in reactive microglia [89]
DUOX2	Structurally similar to DUOX1; maturation depends on DUOXA2 [89]	Regulated by Ca^2+^ binding and DUOXA2 for functional expression [89]	Thyroid gland, gastrointestinal epithelium; implicated in neuroinflammation models (e.g., tau-expressing microglia) [89]

Given the mechanistic and structural diversity among NOX/DUOX isoforms, comprehensive isoform-specific characterization remains essential for the rational development of selective inhibitors. Nevertheless, high potency and target selectivity are crucial requirements for developing compounds involved in chronic conditions such as AD, where off-target effects and systemic redox disruptions must be carefully avoided.

### 2.3. Role of NOX/DUOX in Peripheral Inflammatory Diseases

Peripheral NOX isoforms (NOX1-4) play a central role in amplifying oxidative stress and inflammation across a wide range of pathological conditions. In diabetic retinopathy, chronic hyperglycemia and ischemia/hypoxia trigger NF-κB-mediated upregulation of NOX2, involving both its catalytic core (gp91^phox^) and regulatory subunits p47^phox^ and p67^phox^ [90,91,92,93]. This activation results in an overproduction of superoxide and H_2_O_2_, leading to an oxidative burst marked by the formation of advanced glycation end-products, endothelial cell apoptosis, and microvascular leakage.

Furthermore, NOX4 contributes to chronic tissue remodeling, particularly in renal and pulmonary fibrosis, where sustained H_2_O_2_ release activates pro-fibrotic pathways such as TGF-β signaling. Additionally, NOX4-derived ROS in vascular smooth muscle cells promote the progression of atherosclerotic plaques [75]. In ischemia–reperfusion injury, NOX1 and NOX2 work synergistically to amplify the oxidative burst triggered upon reperfusion, exacerbating tissue damage [94].

Given the intricate interplay between ROS and inflammatory cytokines such as IL-1β and TNF-α [95], therapeutic inhibition of peripheral NOX isoforms has informed strategies for CNS-targeted drug design. However, the clinical failure of non-selective NOX inhibitors in conditions like diabetic retinopathy underscores the imperative for isoform- and tissue-selective approaches to preserve host defense mechanisms while mitigating oxidative injury [96].

To address these challenges, innovative delivery strategies—such as ROS-responsive nanocarriers and BBB permeable systems—are emerging as promising translational tools for targeted antioxidant therapy in AD. For instance, black phosphorus nanosheets combined with methylene blue are capable of reversing neuroapoptosis and neuroinflammation in AD models [97]. Similarly, macromolecular nanoparticles grafted with polyphenols (e.g., resveratrol and oligomeric proanthocyanidins) have been shown to effectively reduce oxidative stress, improving cognitive function in AD mouse models [98].

### 2.4. Role of NOX/DUOX in Neuroinflammation and AD Pathophysiology

**Figure 2:**
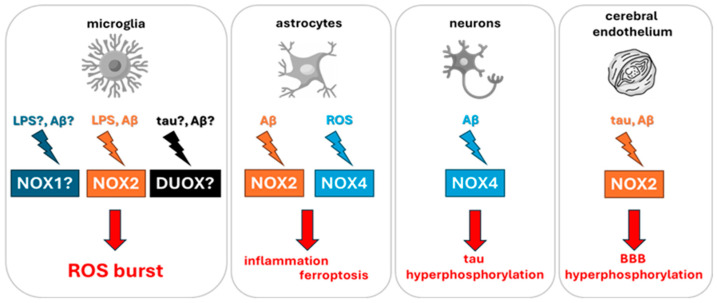
Schematic representation of the main NOX and DUOX isoforms implicated in neuroinflammation and AD. Inflammatory stimuli such as Aβ oligomers and LPS activate NADPH oxidases in CNS-resident cells, including microglia, astrocytes, neurons, and brain endothelial cells. This activation promotes ROS generation, which in turn drives iNOS upregulation, cytokine secretion, BBB disruption, ferroptosis, and tau pathology. Cell-specific isoforms such as NOX2 (microglia and endothelium) and NOX4 (neurons and astrocytes) represent critical targets in AD-related oxidative stress.

Neuroinflammation, a hallmark of AD, involves a complex interplay of microglial activation, pro-inflammatory cytokine release, and BBB disruption. These pathological mechanisms are critically shaped by the activity of NOX and DUOX enzymes, which act as principal sources of ROS within the CNS. In microglial cells, inflammatory stimuli, such as lipopolysaccharide (LPS) or Aβ, trigger Toll-like receptor 4 (TLR4) signaling cascades and NF-κB pathway, leading to the assembly and activation of the NOX2 enzyme complex. This results in the production of superoxide radicals, which are rapidly converted to H_2_O_2_, amplifying oxidative stress. ROS generated through NOX2 activation enhance the expression of inducible nitric oxide synthase (iNOS), promoting elevated NO levels and the subsequent release of pro-inflammatory mediators including TNF-α, IL-6 and IL-1β (Figure 2) [99,100].

ROS-mediated signaling extends beyond microglia, activating the mitogen activated protein kinase (MAPK) and Janus kinase/signal transducer and activator of transcription (JAK/STAT) pathways in astrocytes, further sustaining a pro-inflammatory environment [101,102]. In the context of AD, soluble Aβ oligomers act as potent inducers of NOX2 activity not only in microglia but also in brain endothelial cells. This dual effect leads to degradation of tight-junction proteins, increased BBB permeability, and neurovascular uncoupling, which are early features of AD pathology. Moreover, elevated ROS further impair glucose transporter (GLUT1) function, contributing to the cerebral hypometabolism observed in positron emission tomography studies of early AD (Figure 2) [81,82,83].

In parallel, NOX4 activity in astrocytes promotes lipid peroxidation, leading to iron-dependent cell death via ferroptosis [103]. Instead, neuronal NOX4 exacerbates tau hyperphosphorylation via oxidative inhibition of protein phosphatases (e.g., PP2A), thereby promoting tau aggregation (Figure 2) [104].

Collectively, these NOX/DUOX-driven mechanisms (Figure 1) play a central role in the cascade of events that underlie synaptic dysfunction, neuronal death, and progressive cognitive impairment in AD. Understanding the contribution of these enzymatic sources of ROS to the neuroinflammatory landscape of AD is therefore crucial to inform the isoform-specific characterization of NOX and DUOX enzymes, and to guide the development of targeted inhibitors with sufficient brain penetration and selectivity.

## 3. Isoform-Focused Analysis and Drug Design Implications

### 3.1. NOX Family

Among the NOX family members (Table 2), NOX1 is activated through the assembly with membrane-bound p22^phox^ and cytosolic regulatory subunits such as p47^phox^, p67^phox^, NOXO1, and NOXA1. In neurons, the activation of NOX1 further depends on its nuclear translocation mediated by the GTPase RAC1 [76,77]. This isoform has been extensively studied in peripheral inflammatory models, where it promotes endothelial dysfunction in diabetic atherosclerosis and, together with NOX2, contributes to ischemia/reperfusion injury [75]. However, its role in the CNS remains underexplored.

Notably, in amyotrophic lateral sclerosis (ALS), NOX1-derived ROS oxidize insulin-like growth factor 1 (IGF1) receptors on motor neurons, impairing survival signaling [78]. While NOX1 deletion in SOD1-transgenic mice modestly prolongs animal survival [105], its direct involvement in AD remains untested. Investigating NOX1 in Aβ-overexpressing neuronal cultures and APP/PS1 mouse models (co-expressing mutant amyloid precursor protein and presenilin-1 genes) may uncover novel therapeutic targets relevant to early redox imbalance in AD.

NOX2 consists of the membrane-bound gp91^phox^-p22^phox^ heterodimer and the cytosolic subunits p47^phox^, p67^phox^, p40^phox^, and Rac1/2, whose expression is upregulated by NF-κB in response to inflammatory stimuli [79,80]. Once activated, NOX2 catalyzes a respiratory burst generating superoxide that dismutates to H_2_O_2_, causing damage to lipids, proteins, and DNA [83,106]. In the retina, NOX2-derived ROS are central to the vascular leakage and neuronal apoptosis characteristics of diabetic retinopathy [93,107,108]. In the context of AD, NOX2 assumes an especially deleterious role. Oligomeric Aβ_1–42_ engages microglial TLR4, initiating NOX2 activation and triggering a cascade that compromises BBB integrity. The cascade includes degradation of tight junction proteins, capillary rarefaction, and oxidative DNA damage in endothelial cells [81,82,83,84]. Intriguingly, genetic deletion of NOX2 in AD mouse models preserves neurovascular architecture and cognitive function, even without affecting amyloid plaque deposition [109]. Moreover, clinical observations from ALS patients reveal that lower NOX2 activity in peripheral blood is associated with a striking 7.6-fold reduction in mortality risk [110], enhancing the isoform’s systemic relevance as a redox regulator in neurodegeneration. Taken together, these lines of evidence emphasize NOX2 as not only a central effector of microglial oxidative injury in AD, but also a compelling and actionable target for the development of isoform-selective inhibitors with therapeutic potential.

NOX3 shows a more restricted expression pattern, being primarily located in the inner ear ampulla and selected fetal organs, including the spleen and kidney. In these anatomical sites, NOX3 pairs with p22^phox^ to generate ionic gradients necessary for otoconia formation [85,86]. While NOX3 physiological role is well defined in the vestibular system, its contribution to pathology has been mainly demonstrated in cisplatin-induced cytotoxicity, where NOX3-mediated ROS trigger cochlear hair cell death and chemotherapy-related hearing loss [75,111]. Despite increasing interest in NOX isoforms as contributors to neurodegenerative processes, there is currently a lack of direct evidence implicating NOX3 in AD. Available transcriptomic and proteomic datasets of AD brains have not reported a significant upregulation or redistribution of NOX3 expression, and experimental models of AD have yet to identify functional consequences attributable to this isoform [112,113]. This notable gap in knowledge highlights the importance of future research aimed at determining whether NOX3 could play a previously unrecognized role in AD pathophysiology, particularly in oxidative stress contexts.

NOX4, unlike other isoforms, is constitutively active and is bound to p22^phox^ [73]. This enzyme shows the broadest expression among NOX family members, with particularly high abundance in renal, vascular smooth muscle, endothelial, and neural cells [58,87]. In peripheral tissues, NOX4-derived H_2_O_2_ sustains TGF-β signaling in renal and pulmonary fibrosis and is implicated in diabetic nephropathy and osteoporosis [75,114]. In the brain, NOX4 upregulation in astrocytes facilitates lipid peroxidation through 12- and 15-lipoxygenase activation, ultimately triggering ferroptosis [84]. Neuronal NOX4 has been shown to enhance tau hyperphosphorylation and aggregation by inhibiting protein phosphatases such as PP2A through oxidative modifications [104]. Both conditional knockout and pharmacological inhibition of NOX4 in AD models attenuate tau pathology, synaptic loss, and cognitive decline, identifying this isoform as a primary therapeutic target [104,115]. In line with its broader functional profile, NOX4 and p47^phox^ expression are significantly upregulated in pericytes exposed to high-glucose and angiotensin II, implicating NOX4 in the neurovascular alterations associated with metabolic comorbidities [116]. Given the central role of vascular integrity in AD, these observations highlight the need to further investigate NOX4-mediated redox signaling within the neurovascular unit as a potential contributor to disease progression.

NOX5 is structurally unique among NOX isoforms, as it lacks a rodent ortholog and functions independently of p22^phox^. Enzyme activation occurs through Ca^2+^ binding to EF-hand motifs and phosphorylation. NOX5 is highly expressed in human testis, spleen, and lymphoid tissues. Recently the resolution of its crystal structure has opened new possibilities for the design and development of new compounds [88]. Despite the lack of in vivo CNS data, preclinical systems such as humanized mice or brain organoids will be essential to assess the role of NOX5 in the brain and its relevance in AD. Current literature links NOX5 to cardiovascular hypertrophy, renal injury, and oncogenesis [74], but its implications for neurodegeneration remain largely speculative and warrant further investigation.

**Table 2:** Comparative overview of NOX and DUOX isoforms highlighting their physiological roles, pathological implications, and specific involvement in neurodegeneration and AD.

Isoform	Physiological Role	Pathological Implications	Neurodegeneration Evidence	AD-Specific Relevance
NOX1	Redox signaling in epithelium and vasculature [75]	Endothelial dysfunction, I/R injury, vascular inflammation [75]	Impaired IGF1 signaling in ALS models [78]	Lacking direct evidence; further investigation needed [105]
NOX2	Host defense, phagocyte ROS production [81,82,83,84]	BBB disruption, neuronal apoptosis, vascular injury [81,82,83,84]	Strong evidence in ALS, PD, and AD models [81,82,83,84]	Activated by Aβ_1–42_; promotes neuroinflammation and cognitive decline [81,82,83,84]
NOX3	Otoconia formation in the inner ear [85,86]	Cisplatin-induced ototoxicity [85,86]	Not yet characterized	No evidence; potential role under oxidative stress to be investigated
NOX4	Constitutive ROS production for cellular homeostasis [73]	Fibrosis, tau hyperphosphorylation, ferroptosis [87,104]	High expression in CNS; implicated in tauopathy [104,117]	Strong evidence; pharmacological inhibition improves cognition in animal models [117]
NOX5	Ca^2+^-dependent ROS generation (no rodent ortholog) [88]	Cardiovascular and renal injury [88]	No CNS data available	Speculative; requires study in humanized models/organoids
DUOX1/2	H_2_O_2_ for thyroid hormone synthesis [89,118]	Congenital hypothyroidism, epithelial inflammation [89,118]	Upregulated by tau in fly models; M2000 reduces DUOX in rats [117]	Emerging evidence; underexplored therapeutic target in AD

### 3.2. DUOX Family

Beyond the well-known NOX isoforms, scientific attention is increasingly being directed toward the dual oxidases (Table 2), which possess distinctive structural features and emerging functional relevance within redox biology. Their involvement in neuroinflammation and neurodegeneration is gaining recognition and merits hereby further exploration.

DUOX1 and DUOX2 are structurally characterized by extracellular peroxidase-like domains and intracellular EF-hand Ca^2+^ binding motifs. Their proper localization and activation require specific maturation factors, DUOXA1 and DUOXA2, respectively. Physiologically, they produce H_2_O_2_ to support thyroid hormone biosynthesis via thyroperoxidase activity [89], and DUOX2 mutations cause congenital hypothyroidism [118]. In neurodegeneration, DUOX expression is induced by Aβ_42_ and tau, amplifying microglial oxidative stress and neuronal damage [38,119].

Notably, *Drosophila* models expressing tau but not Aβ_42_ show increased DUOX mRNA levels [119], supporting isoform-specific transcriptional regulation. Strikingly, the NSAID M2000 has demonstrated the ability to downregulate DUOX expression and reduce amyloid burden in AD rat models, improving their cognitive performances [120]. These findings suggest DUOXs may represent underexplored but pharmacologically relevant targets, particularly for medicinal chemists seeking to mitigate neuroinflammation without compromising peripheral endocrine function. Despite these promising observations, current knowledge on DUOX1 and DUOX2 in neurodegeneration remains fragmentary, and their roles in AD are far from elucidated. Importantly, these enzymes are highly conserved from *Drosophila* to human, supporting the translational relevance of findings obtained in invertebrate models [121]. Future research should prioritize the use of mammalian systems to comprehensively assess the pathogenic and therapeutic implications of DUOX activity in AD.

## 4. NOX Inhibition and Its Therapeutic Potential in AD

### 4.1. Broad, Non-Selective Flavoprotein Inhibitors and Early Tool Compounds

The development of NOXs inhibitors has been a topic of substantial interest, seeing their potential therapeutic relevance in the context of pathologies where the physiological redox state is altered. However, NOX inhibitors have historically been plagued by selectivity issues, which have significantly hindered the possibility of studying their precise effects in physiopathology and obviously the possibility of clinical translation of medicinal chemistry and pharmacology efforts. This is especially important considering the varied landscape of NOX isoforms, which makes it imperative to find selective inhibitors in order to understand each isoform’s specific physiopathological role and potential druggability [74,122].

There appears to be an inherent difficulty in investigating NOX inhibition. This is intimately linked to the products of their enzymatic activity, which have been determinant for the availability of bioassays for their investigation. Enzymatic activity assays are usually based on readouts of superoxide anion or H_2_O_2_ produced by NOXs, but such assays are prone to interference and detection of false positives. Indeed, NOXs are not the only source of ROS, with other enzymes such as xanthine oxidase (XO), cytochrome P450 oxidases (CYP450), lipoxygenases, monoamino oxidases (MAO), uncoupled nitric oxide synthase (eNOS), also implied in their production. These interferences are especially relevant in the setting of cell-based assays, in which whole cells (generally primary neutrophils or derived cell lines such as HL60) are made to overexpress the NOX isoform of interest. Moreover, the presence of antioxidant groups in a putative NOX inhibitor can also interfere with ROS production readouts [74].

These technical limitations, which have characterized the first decade of NOX study, lead to the first generation of NOX inhibitors: these are nonselective pan-inhibitors, which in many cases can also interact with other proteins implied in redox metabolism. This has made them sometimes useful as investigational tools, but has significantly hampered their viability in clinical settings.

**Figure 3:**
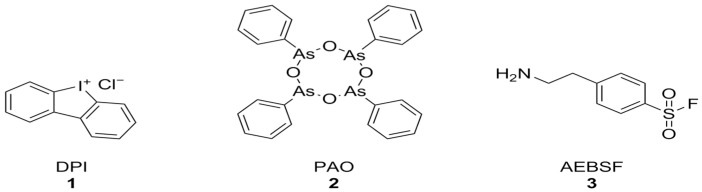
Structural formulas of DPI, PAO, and AEBSF.

Diphenyleneiodonium chloride (DPI) [123], phenylarsine oxide (PAO) [124], and 4-(2-aminoethyl)-benzenesulfonyl fluoride (AEBSF) [125] (Figure 3) were among the first identified NOX inhibitors. These compounds, however, have plenty of off-target effects: PAO is also a tyrosine phosphatase inhibitor as well as a reducing agent, ABF is a non-specific serine protease inhibitor, DPI can interact with a large number of flavoproteins (including XO and CYP450), and all of these compounds display relatively low potencies and no selectivity between NOX isoforms [74]. Nonetheless, DPI has shown protective effects against Aβ-induced neurotoxicity in primary hippocampal neurons that were synergistic with the activation of mitochondrial potassium channels by diazoxide [126].

**Figure 4:**
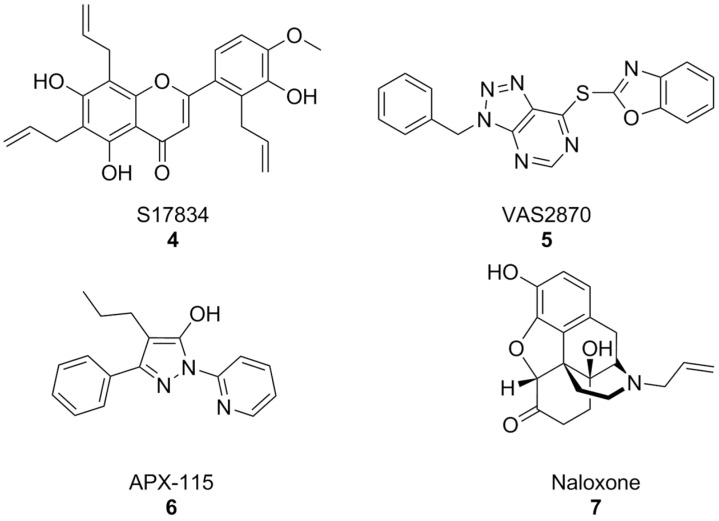
Structural formulas of synthetic, non-selective NOX inhibitors.

In parallel, other early synthetic inhibitors were identified. Compound S17834 (Figure 4) is a synthetic flavonoid originally characterized as an inhibitor of adhesion molecule expression (e.g., VCAM1), whose NOX inhibition was proposed as its major mechanism of action. While S17834 shows preferential activity toward NOX compared to XO, no information is available on its isoform selectivity or potential utility in neurodegenerative disease models [127]. Moreover, like many other phenolic compounds, S17834 appears to be much more potent as a stimulant of AMP-activated protein kinase, overshadowing its activity toward NOX [128].

Early high throughput screening efforts resulted in the identification of triazolopyrimidine VAS2870 (Figure 4) as a selective inhibitor of NOX2 [129,130]. However, subsequent studies disproved its selectivity [131,132] and brought to light several issues for its application which were essentially related to its capacity to alkylate the thiol groups of cysteine residues of a large variety of proteins [133]. While most studies have focused on the potential utility of VAS2870 and its triazolopyrimidine congeners in the context of cardiovascular disease [134], VAS2870 proved capable to protect astrocytes from staurosporin-induced toxicity [135].

Of the many other examples of synthetic pan-NOX inhibitors that have been discovered, many have been called into question as potential assay interferents or have shown substantial off-target effects, and none has been tested in the context of AD models, most notably pyrazole derivative APX-115, also known as isuzinaxib (Figure 4). This compound showed a good safety profile and promising pharmacokinetic properties in Phase I [136], and has reached Phase II clinical trials for diabetic kidney disease [137]. A Phase II clinical trial of APX-115 on mild to moderate COVID-19 patients was instead terminated, although reasons for this are yet unknown [138].

Opioid antagonist naloxone (Figure 4) has been reported as a NOX2 inhibitor and was successfully shown to be a neuroprotective agent in a mouse model of Parkinson’s disease. However, its effects on other NOX isoforms were not reported. An in-depth description of many of these substances and their pharmacology has been carried out in a recent perspective article [65], to which we refer the interested reader.

Collectively, these first-generation inhibitors served primarily as tool compounds to reveal the contribution of NOX-derived ROS to neurotoxicity, yet their lack of selectivity precludes their use as robust pharmacological probes for NOX isoform function in AD.

### 4.2. Compounds with Dual Antioxidant and NOX-Modulatory Actions

A second category includes compounds historically described as NOX inhibitors but whose predominant actions involve antioxidant activity, redox cycling, or modulation of cellular redox responses rather than direct inhibition of the catalytic core of NOX enzymes.

**Figure 5:**
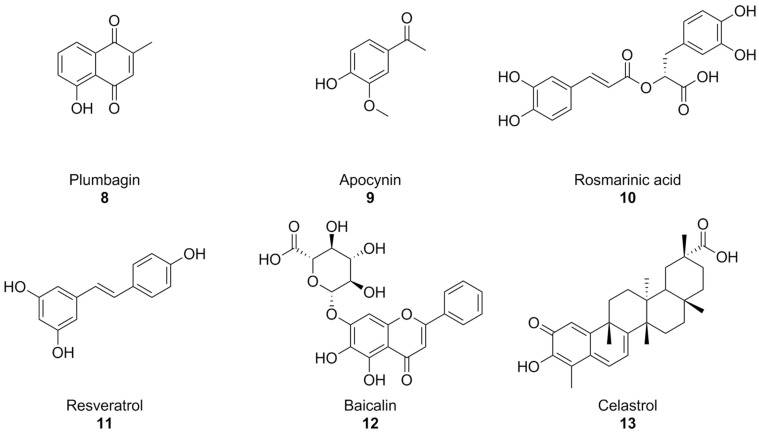
Structural formulas of natural phenolic compounds with reported activity against NOXs.

With the turn of the millennium, some natural compounds, such as apocynin (a constituent of Himalayan medicinal plant *Picrorhiza kurroa*) [139] and plumbagin [140] (Figure 5) were also investigated as NOX inhibitors. Similar to the previously seen synthetic small molecules, these compounds exhibited very low potencies. Indeed, it appears that the products of the oxidative metabolism of apocynin are much more potent NOX inhibitors than their precursor (with IC_50_ values in the nanomolar range, compared to the ~300 µM reported for apocynin), while apocynin acts more like an antioxidant [141,142]. in regard to isoform selectivity, no data have been reported for apocynin and its derivatives [74,141], while the best-known effects of plumbagin are on NOX-4 [140].

Plumbagin and apocynin are per se radical scavengers, casting doubts over their capacity to truly block ROS formation via NOX inhibition [74,142], and plumbagin in particular is a known genotoxic and mutagenic substance [143,144]. This is especially important to contextualize some recent reports of the activity of plumbagin as a neuroprotective agent in mouse models of AD [145,146]. Reports on the neuroprotective and anti-AD effects of apocynin are controversial: while chronic treatment with this substance was shown to reduce amyloid plaque size and microglial recruitment in the hAPP(751)SL mouse model of AD, it did not result in significant cognitive improvements [147]. In a different mouse model (Tg19959), apocynin showed little to no effect also on amyloid plaque size and microglial involvement [148].

In moderately aged rat, a red grape polyphenol extract was found to lower superoxide production in the liver. This was linked to NOX inhibition, but no information regarding the precise polyphenolic composition of this extract was provided [149]. Rosmarinic acid (Figure 5), a polyphenol present in plants of the *Lamiaceae* and *Boraginaceae* families, inhibitied platelet aggregation with a NOX-dependent effect in an in vitro model of Aβ aggregation [150], while resveratrol (Figure 4), found in grapes and peanuts, was shown to inhibit the expression of these enzymes in BV-2 microglial cells activated by oligomeric Aβ [151]. Similarly, baicalin, a flavonoid derived from traditional chinese medicinal plant *Scutellaria baicalensis*, was recently found to also lower NOX2 and NOX4, as well as oxidative stress damage in mouse hearts treated with isoproterenol [152]. Again, it must be remarked that no studies were conducted on the specificity of these substances as NOX inhibitors, and that their intrinsic antioxidant capacity might interfere with attempts to evaluate them as such.

Celastrol (Figure 5), a bioactive phenolic compound found in traditional Chinese medicinal plant *Tripterygium wilfordii*, was also reported as a pan-NOX inhibitor with slight selectivity towards NOX1 and 2 (IC_50_~0.5 µM) compared to NOX4 and NOX5 (IC_50_~3 µM). This selectivity was consistent with data regarding celastrol’s capability to interact with p47^phox^ by covalently modifying a cysteine residue and preventing its association with p22^phox^. There exists a good amount of literature surrounding the potential anti-AD effects of celastrol on learning and memory in AD mouse models [153,154,155,156]; however, evidence of its actual effectiveness is controversial, and a recent paper titled Celastrol Attenuates Learning and Memory Deficits in an Alzheimer’s Disease Rat Model on this matter was retracted.

### 4.3. Modern, Isoform-Preferential NOX Inhibitors

NOX2, expressed in the microglia but also in cerebral vasculature and, to a lower extent, in neurons, is the main target for anti-AD drug development efforts [157]. Advances in assay development, structural modeling, and medicinal chemistry have recently enabled the emergence of more selective NOX inhibitors with improved isoform preference, providing more reliable tools to recognize individual NOX contributions in AD.

**Figure 6:**
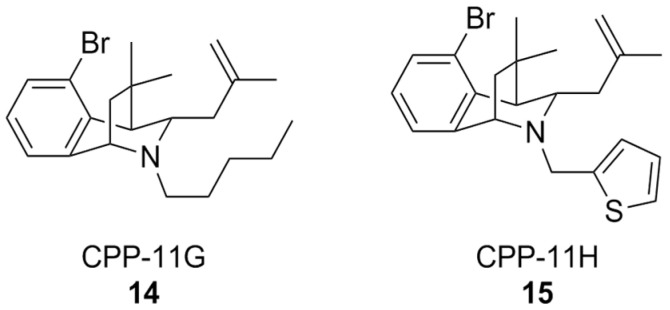
Structural formulas of bridged tetrahydroisoquinolines CPP11-G and CPP11-H.

A classical approach to investigate NOX isoform selectivity is to carry out a preliminary screening for ROS production in cell lines stably expressing the NOX isoform of interest, and subsequently in a cell free setting using the purified membrane-bound protein from a cell lysate. Generally, chemiluminescent or fluorescent probes are used for these assays. Such a methodology has led to the discovery of bridged tetrahydroisoquinolines as NOX 2 inhibitors, namely as CPP-11G and CPP-11H (Figure 6), bearing thiophene or n-pentane on the scaffold’s nitrogen atom. These structural characteristics were linked to their relative inactivity at NOX1, NOX4, and NOX5 compared to other congeners over the course of a structure-activity relationship (SAR) study [158]. Further in silico analysis showed that the selectivity of these compounds towards NOX2 could disrupt interactions between p22^phox^ and p47^phox^, which is crucial for NOX2 activity. In vitro studies of their mechanism of action showed that they were capable of inhibiting membrane translocation of p47^phox^. Downstream of NOX2 inhibition, it was finally shown that both CPP11-G and CPP-11H could ameliorate endothelial inflammation in human aortic endothelial cells (HAECs) as well as in vivo in mice [159]. These compounds are currently under evaluation as potential neuroprotective agents [157], based on their capability to cross the BBB and inhibit NOX2 assembly in brain tissue [160].

**Figure 7:**
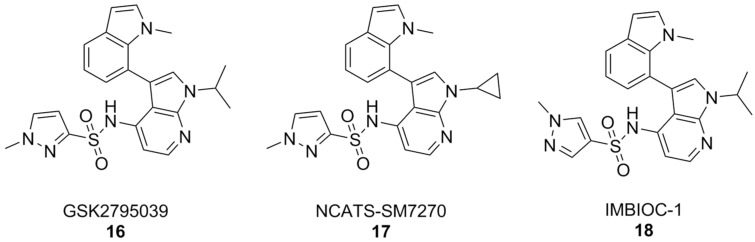
7-Azaindole NOX2 inhibitors.

By and large, the most substantial results were obtained by the 7-azaindole class of NOX2 inhibitors (Figure 7). Compounds of this class, such as GSK2795039, first came out of a HTS campaign by the pharmaceutical company Glaxo Smith Kline. With potency in the low micromolar range and selectivity towards this enzyme isoform, GSK2795039 acts by competing with NADPH for its binding site on NOX2, thereby lowering the production of ROS in microglia [161,162,163]. In vivo, GSK2795039 can ameliorate neurological symptoms in a mouse model of traumatic brain injury [110]. It can reverse Aβ-related glucose hypometabolism and hippocampal neuron hyperactivity, as well as improve animal behavior in a mouse model of AD, as well as improve animal behavior in a mouse model of AD [68].

Critically, GSK2795039 has poor pharmacokinetic properties, chiefly low bioavailability and a high clearance rate [164], which have led to the development of NCATS-SM7270. The variations on positions 1 and 3 of the 7-azaindole scaffold of GSK2795039, namely the replacement of the 1-isopropyl group with a cyclopropyl and the 3-methylindoline with a 3-methylindole, have led to a compound that is both more potent and with better pharmacokinetic properties than its forerunner, while keeping a good degree of selectivity towards NOX2 [165]. These similarities were translated in vivo, where NCATS-SM7270 was also capable of alleviating traumatic brain injury-induced damage to cortical neurons in a mouse model, although no studies on AD mouse models have been carried out yet [165]. GSK2795039 was instead studied in the intracerebral Aβ administration mouse model of AD, where it prevented microglial activation and neuroinflammation as well as oxidative pathology. Overall, this resulted in inhibition of cognitive and neuropsychiatric symptoms [166].

The synthesis of GSK2795039 and NCATS-SM7270 was recently optimized, affording a synthetic route that can be scaled to yield gram quantities of 7-azaindole compounds. Over the course of this study, an additional 7-azaindole derivative was obtained (IMBIOC-1) (Figure 7). The three 7-azaindoles were found to have very similar binding poses to NOX2 in a molecular docking study, with the new derivative IMBIOC-1 showing the highest affinity to this target. Moreover, all three compounds showed no significant intrinsic cytotoxicity on human microglia HMC3 cells, and fully counteracted Aβ-related cytotoxicity by lowering oxidative stress. Molecular docking studies indicate that all three compounds adopt similar poses to interact with the NOX2 binding pocket, but the indole group of IMBIOC-1 forms hydrophobic interactions with a different residue compared to the other two compounds (324Tyr instead of 339Pro). All three compounds form a hydrogen bond with the basic sidechain of residue 73Arg, however GSK2795039 and NCATS-SM2720 contract this interaction through their sulfonyl oxygen, while IMBIOC-1 forms it through a nitrogen atom on the pyrazole ring [167].

**Figure 8:**
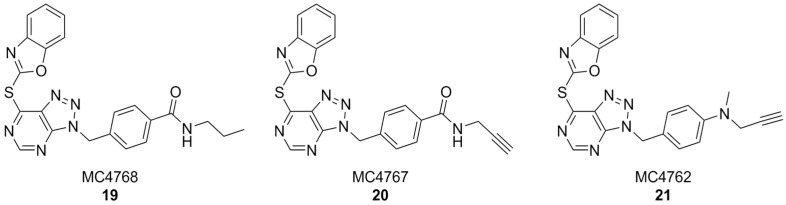
VAS2870 derivatives MC4768, MC4767, MC4762.

In another interesting approach, benzyl-triazolopyrimidine derivatives based on the structure of VAS2870 were synthesized and tested on *Cylindrospermum stagnale* NOX5 as a preliminary screening assay. At first, the 2-thiobenzoxazole leaving group of this compound was replaced with smaller substituents, such as hydroxy, mesilate, chloro- or amino groups, but all resulting compounds were inactive. This proved the hypothesis that the 2-thiobenzoxazole is essential to covalently bind an essential cystein residue of the enzyme. Replacing the 2-thiobenzoxazole with trapping substituents such as acrylamides or carbamates by using a piperidine or piperazine linker to connect them to the benzyl-triazolopyrimidine core still resulted in compounds with much lower activity than VAS2870. Better results were obtained with compounds bearing a *p*-propylamide, *p*-propargylamide or *p*-propargylamine groups linked to the benzyl moiety of VAS2870, which kept the 2-thiobenzoxazole group and were thus capable to covalently bind the target enzyme (respectively, MC4768, MC4767 and MC4762, Figure 8). Further bioassays on purified, activated NOX2 and on NOX2-overexpressing cells confirmed the promising activity of propargylamino derivative MC4762 with IC_50_ = 0.155 µM (purified NOX2) and 0.135 µM (in cellulo). At the same time, MC4762 showed promising selectivity toward other NOX isoforms, and was also active as an inhibitor of monoamino oxidase B (MAOB), with a very similar potency compared to NOX2 (IC_50_ = 0.182 µM). These results were confirmed in microglial BV2 cells. Indeed, MC4762 is a highly attractive multi-target directed ligand (MTDL) for further studies in the AD field due to its activity at NOX2 and MAOB, an additional potential target for AD therapy [168].

### 4.4. Indirect Approaches Targeting Regulatory Subunits and Protein–Protein Interactions

Given the modular structure of NOX complexes, indirect inhibition strategies that interfere with enzyme assembly and activation represent an alternative to catalytic-site targeting. These approaches are particularly relevant for NOX2, whose activation requires the coordinated association of cytosolic and membrane-bound subunits [158].

It is easy to see how the application of protein–protein interaction (PPI) assays to the screening and characterization of new NOX inhibitors has substantially pushed forward this field of investigation. Since the production of ROS is not involved in this kind of assay, there is little to no interference by radical scavenging groups, so even compounds with intrinsic antioxidant activity can be claimed as hits. The aforementioned study of apocynin derivatives is an example of this approach, where the interaction of immobilized biotin-p22^phox^ and his-p47^phox^ was studied via ELISA in the presence or absence of apocynin and its oxidized derivatives [141]. Although that study did not focus on a specific NOX isoform, using vascular-endothelial cell derived NADPH oxidase p47^phox^, it showed that PPI assays can be more easily targeted to specific NOX isoforms. In fact, their availability has made it possible to study the specificity of known and novel NOX2 inhibitors.

Earlier on, the Pagano group had already exploited the disruption of the p22^phox^ and p47^phox^ interaction with the development of a NOX2-derived peptide combined with the cell-permeation inducing HIV-TAT sequence, yielding the NOX2ds-tat peptide, which, among several other effects, was very recently found to improve the symptoms of traumatic brain injury in mice [169,170].

**Figure 9:**
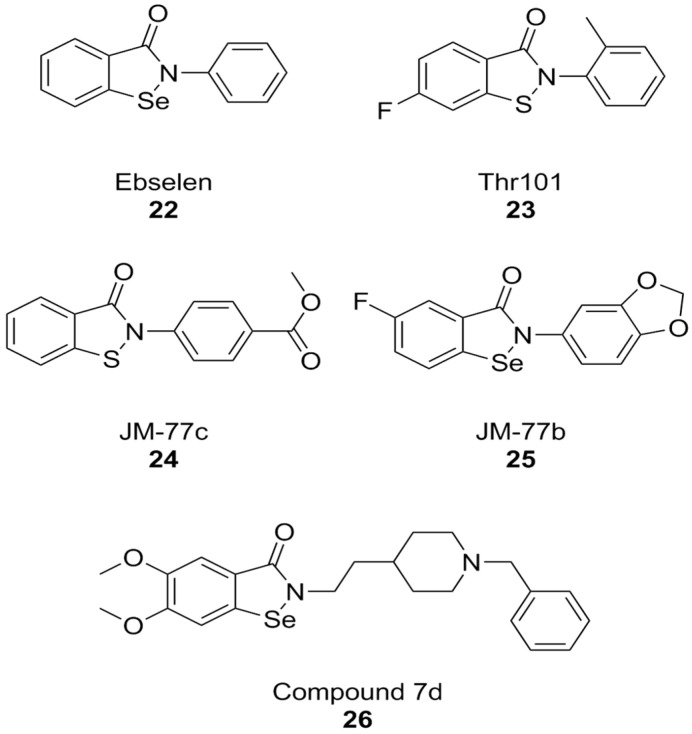
Structural formulas of ebselen and selected derivatives.

A PPI investigation approach led to the identification of ebselen (Figure 9), an organic selenium-containing compound previously known for being a glutathione peroxidase mimetic and a ROS scavenger, as a NOX inhibitor. In this case, a fluorescence polarization high throughput screening (HTS) assay was developed for NOX2 and applied to a chemical library. Ebselen (NOX2 IC_50_ = 0.3 µM in the FP assay and 0.6 µM in the cell-free enzymatic assay), as well as its congeners that were designed and synthesized over the course of this study, showed substantial selectivity towards NOX1 and NOX2 in subsequent cell-based studies in human neutrophils (which constitutively express high levels of NOX2 only), HEK cells stably expressing human NOX1, and HEK cells transiently expressing NOX5. Another compound coming out of this study was Thr101 (Figure 8), a sulfur-containing analog of ebselen, which moderately inhibited NOX2 subunit binding and activity (IC_50_ = 4 µM in both assays), while other analogs in which selenium was replaced with oxygen, carbon, nitrogen or sulfonamine showed no activity at all. The only structural modifications that resulted in active compounds regarded the substituents on the two aromatic rings other than the selenium- or sulfur-containing one. Among sulfur-containing analogs, the best results were obtained by a benzoisothiazolone analog containing a p-COOCH_3_ group (JM-77c, NOX2 IC_50_ = 0.8 µM) (Figure 9). Bulkier substituents on this position showed progressively worse activities. A similar substitution on ebselen improved binding potency only modestly, while the best results in this class were obtained by a compound with a fluoro-substitution on the left side ring, and with the fusion of a 1,4-dioxole ring to the right-hand aromatic moiety (JM-77b) (Figure 9) [171].

The therapeutic potential of ebselen in AD therapy has been the object of several recent studies. At high concentrations (10.94 µM) this compound could improve cognitive parameters in triple-transgenic AD (3 × Tg-AD) mice but was also toxic. Further studies have shown that even lower concentrations can lead to behavioral improvements, Aβ clearance, and synaptic damage recovery in the same mouse model. At the same time, ebselen caused improvements in mitochondrial function in both mouse neuroblastoma N2a-SW cells and in AD mouse brain tissue [172]. Similarly, ebselen was capable to reverse neurodegenerative markers (memory impairment, oxidative stress in the hippocampus, and apoptosis) in streptozotocin-induced sporadic AD mouse model [173]. While among NOX isoforms, ebselen is somewhat selective towards NOX1 and NOX2, much like celastrol, its anti-AD effects cannot be attributed to NOX inhibition alone. The literature shows that ebselen can inhibit human acetylcholinesterase (AChE), the most well-established molecular target for the therapy of AD [174]. Moreover, its structure was used as a starting point for the development of hybrid compounds with clinically relevant anti-AD AChE inhibitor donepezil, obtaining potent inhibitors of these enzymes such as compound 7d (hAChE IC_50_ = 0.097 µM). While this compound was also characterized as a modest butyrylcholinesterase inhibitor and as a ROS scavenger, no data are available regarding its activity as a NOX inhibitor [175].

**Figure 10:**
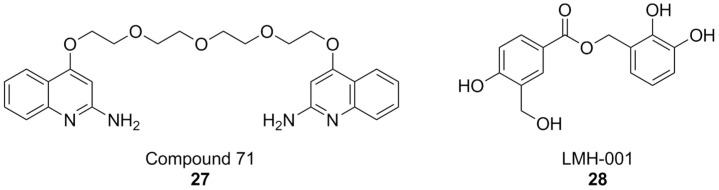
Structural formulas of other PPI-directed NOX2 inhibitors.

The discovery of ebselen inspired an HTS campaign using a library of 2500 chemical fragments, which was carried out by fluorescence polarization and thermal shift assays. Hit validation was performed by surface plasmon resonance. Two quinolone fragments were then identified, and related derivatives were synthesized, yielding compounds like 71 (Figure 10), which showed Ki values in the micromolar range. Although promising, the affinity of compound 71 was insufficient to warrant more in-depth biological tests for its potential therapeutic utility. While the authors claim NOX2 selectivity, no experiments have been conducted to rule out the possibility of off-target effects [176].

It is worth noting that, while the study of PPI at the interaction of p22^phox^ and p47^phox^ has accelerated NOX research, it has not completely eliminated the risk for false positives: as an example, the results first obtained by LMH001 as a NOX2 inhibitor of this kind [177], were not replicated in subsequent follow-up studies [178]. Moreover, compound selectivity against other NOX isoforms is still drastically underreported, even in current literature, putting into question the results of a large amount of research work that went into the development of NOX2 inhibitors [94,179,180,181].

## 5. The Future of NOX Inhibitors for AD Therapy

The development of NOX inhibitors is a burgeoning field of research that holds considerable promise for the future, and NOX2 inhibitors, in particular, have begun to reach a sufficient level of maturity, as we saw in the previous sections. However, while NOX2 is the most strongly implicated NOX isoform in AD pathology, other NOX isoforms, chiefly NOX4 and possibly DUOX1-2, could also play a role, and thus be potential targets for its resolution.

SAR studies regarding NOX inhibition, whether specific or not, are still rare in the literature, and it is also rare to find information regarding the binding modes of inhibitors to their specific NOX isoform or regulatory subunit. We have summarized the information compiled in the previous section in the light of compound chemical class (Table 3).

**Figure 11:**
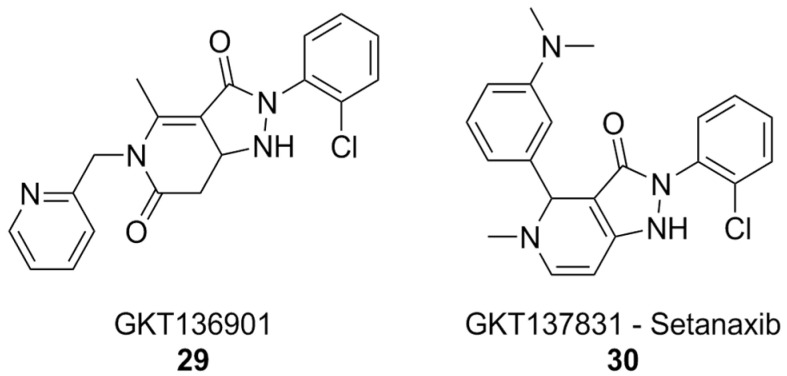
NOX1/4 inhibitor setanaxib.

The pyrazolopyridinedione class of NOX inhibitors, which includes compounds that have reached clinical trials, holds significant promise in this regard. The forerunner of this class of compounds, GKT136901 (Figure 11), showed selectivity towards NOX4 and NOX1 compared to NOX2. SAR studies and lead optimization eventually yielded compound GKT137831, also known as setanaxib (Figure 11). Setanaxib’s promising pharmacodynamic and pharmacokinetic profiles have led to its investigation in clinical settings for the treatment of multiple diseases. This compound passed an open-label Phase I [182] clinical trial, and showed good results in Phase II for the treatment of primary biliary cholangitis [183]. Phase II trials for the treatment of idiopathic pulmonary fibrosis were completed, but no results have been published yet [184]. Results of another Phase II study of setanaxib, this time in the context of nephropathy in type 2 diabetes, were ambiguous: the treatment did not reach the primary efficacy endpoint, mesaured as albuminuria reduction, but still reached secondary endpoints like inhibition of the renin–angiotensin–aldosterone system [185]. Finally, a Phase II study of setanaxib in combination with pembrolizumab for the therapy of recurring or metastatic squamous cell carcinoma of head and neck was also completed, but the effect of setanaxib was negligible compared to that of pembrolizumab alone [186].

In vitro, setanaxib was capable of alleviating the release of ROS by activated microglia, suggesting that it or similar molecules could be beneficial for the treatment of pathologies involving neuroinflammation, such as AD itself. Unfortunately, off-target effects and possible sources of interference, such as direct scavenging activity, plague setanaxib as well, and it has recently been suggested that its mechanism of action might not be so straightforward as just inhibition of NOX1/4, limiting the significance of currently available data [122].

The current picture of NOX inhibitors is that of a growing, but still immature field of pharmaceutical investigation. Indeed, the lack of clinically available NOX inhibitors means, among other things, that there is little data regarding the potential downsides of this kind of therapy. The existence of pathologies like chronic granulomatous disease, a genetic disorder linked to defects in NOX2 or its regulatory subunits, is evidence that a modicum of NOX2 activity is essential for proper immune function, as total abolition of this enzyme results in a catastrophic series of effects such as impaired innate immunity, but also hyperinflammation and autoimmune disease. Thus, care will have to be taken when considering to expose patients to high doses of NOX inhibitors over long stretches of time. Prophylactic co-administration of antibiotics could be an option for older AD patients, whose advanced disease could require chronically high doses of NOX inhibitors resulting in immunodepression [187]. It also remains to be seen what the potential impairment of Aβ clearance by the microglia, which could be caused by NOX2 inhibition, would mean in terms of disease progression.

The bioassays for NOX inhibition have shown significant improvement over the last decade, with novel, bioluminescence-based PPI assays becoming widely available in recent years [75]. While these assays have been mainly applied to NOX2 and NOX4 interactions with p22^phox^, they could potentially also be developed for DUOX1 and DUOX2 interactions with the respective maturation factors DUOX1A and DUOX2A, leading finally to the discovery of selective compounds for these poorly characterized enzymes, and to an elucidation of their role in a variety of pathologies, including, crucially, AD.

## Figures and Tables

**Table 3:** A summary of data regarding each compound reported in Section 4, divided by chemical class.

Chemical Class/Structural Core		Inhibitor	NOX Isoforms Targeted	Known Off-Targets	Evidence in AD-Relevant Models	Testing Stage	References
Diphenyleneiodonium		DPI (**1**)	Pan-NOX	Multiple flavoproteins	Protection from Aβ-induced neurotoxicity in primary hippocampal neurons	In vitro	[74,123,126]
Organoarsenical		PAO (**2**)	Pan-NOX	Tyrosine phosphatases	None	In vitro	[74,124]
Arylsulfonyl fluoride		AEBSF (**3**)	Pan-NOX	Serine proteases	None	In vitro	[74,125]
Triazolopyrimidine		VAS2870 (**5**)	Pan-NOX	Cys alkylation across proteins	None	In vitro	[129,130,131,132,133,134,135]
		MC4768 (**19**)	NOX2	Not reported	None	In vitro	[168]
		MC4767 (**20**)	NOX2	Not reported	None	In vitro	
		MC4762 (**21**)	NOX2	MAO-B inhibitor	Potential multitarget inhibitor of NOX2 and MAO-B in microglia	In vitro	
Pyrazolylpyridine		APX115 (**6**)	Pan-NOX	Antioxidant	None	Phase II ongoing	[136,137,138]
Morphinan		Naloxone (**7**)	Reported NOX2	µ-opioid receptor	None	Approved drug	[65]
Phenolic	Quinone	Plumbagin (**8**)	NOX4	Genotoxicity, mutagenicity, redox cycling	Neuroprotective effects in AD mice	In vivo	[74,140,142,143,144,145,146]
		Celastrol (**13**)	NOX1/2 > NOX4/5	Covalent p47^phox^ interactions; multiple proteins	Improved memory in AD mice (retracted)	In vivo	[153,154,155,156]
	Methoxy cathecol	Apocynin (**9**)	Weak/unclear	Antioxidant; redox enzymes	Conflicting data	In vivo	[74,139,141,147,148]
	Polyphenol	Rosmarinic acid (**10**)	Unclear	Antioxidant	Inhibits platelet adhesion in Aβ aggregation model	In vitro	[150]
		Resveratrol (**11**)	Unclear	Antioxidant	Inhibits NOX in BV2 cells activated by Aβ	In vitro	[151]
		LMH-001 (**28**)	NOX2 (disputed)	Antioxidant	None	In vitro	[176,177]
	Flavonoid	S17834 (**4**)	No data	AMPK activator	None	In vitro	[127,128]
		Baicalin (**12**)	NOX2/4 (putative)	Antioxidant	None	In vivo	[152]
Bridged tetrahydroisoquinolines		CPP11-G (**14**)	NOX2	Minimal reported	Studies ongoing	In vivo	[157,158,159,160]
		CPP11-H (**15**)					
7-Azaindole		GSK2795039 (**16**)	NOX2	Few data	Improved metabolism, neuronal activity and behavior in AD mice	In vivo	[68,110,161,162,163,164,166,167]
		NCATS-SM7270 (**17**)	NOX2	Few data	None	In vivo	[165,167]
		IMBIOC-1 (**18**)	NOX2	Few data	Restored viability of Aβ-treated microglia	In vitro	[167]
Organoselenium		Ebselen (**22**)	NOX1/2	AChE inhibition; antioxidant	Improved cognition & reduced pathology in AD mice	Phase II complete	[171,172,173,174]
Ebselen analogs		Thr101 (**23**)	NOX1/2	Unknown	None	In vitro	[171]
		JM-77b (**24**)					
		JM-77c (**25**)					
		Compound 7d (**26**)	No data	AChE inhibition	None	In vitro	[175]
Quinolone fragment dimer		Compound 71 (**27**)	NOX2	Few data	None	In vitro	[176]

## Data Availability

No new data were created or analyzed in this study.
